# Supplementary material for: Compliance towards WHO recommendations on antenatal care for a positive pregnancy experience: Timeliness and adequacy of antenatal care visit in Sub-Saharan African countries: Evidence from the most recent standard Demographic Health Survey data
Source: PLoS One. 2024 Jan 25;19(1):e0294981. doi: 10.1371/journal.pone.0294981 (PMC10810464; doi:10.1371/journal.pone.0294981)
Supplement: Attachment — Submitted filename: Response to Reviewers.docx [file pone.0294981.s001.docx]

**AUTHORS’ RESPONSE TO ACADEMIC EDITOR AND REVIEWERS**

1. **Author’s Response To Academic Editor**

**Dear: Hiwot Yisak Dawid, MPH, (Academic Editor)**

We thank you for a thorough reading and constructive comments and suggestions on our manuscript and for the opportunity to revise and resubmit. We are pleased to submit the revised research article “**Compliance towards WHO recommendations on antenatal care for a positive pregnancy experience: Timeliness and adequacy of antenatal care visit in Sub-Saharan African countries: Evidence from the most recent standardized Demographic Health Survey data**” for your consideration in the special collection of Plos One. On the following pages, you will find our responses to the questions raised by the esteemed editor and a reviewer. On behalf of my co-authors, I thank you for your consideration of this resubmission. We appreciate your time and look forward to your response.

Sincerely,

Aklilu Habte(MPH)(corresponding author)

[**aklilihabte57@gmail.com**](mailto:aklilihabte57@gmail.com)

**Journal requirement**

**Requirement 1.** Please ensure that your manuscript meets PLOS ONE's style requirements, including those for file naming.

**Response:** Thank you for the reminder. But, from the start of the submission, we tried to follow the journal's manuscript submission guidelines, and we have now double-checked all of the requirements.

**Requirement 2.** Please check the tables "Table 1: List of the individual- and community-level factors that affect the timing and adequacy of ANC, in SSA countries 2016-2021" "Table 1: Description of the SSA countries included in the analysis with their respective sample size, 2016-2021".

**Response**: We appreciate your thorough review. Of course, misplacing the tables was a mistake, which we have now corrected, and highlighted in the "Explanatory variables" section of the "Revised manuscript with track changes," Line 173, Page 7.

**Requirement 3.** In your revised cover letter, please address the following prompts:

**3.1.** If there are ethical or legal restrictions on sharing a de-identified data set, please explain them in detail (e.g., data contain potentially sensitive information, data are owned by a third-party organization, etc.) and who has imposed them (e.g., an ethics committee). Please also provide contact information for a data access committee, ethics committee, or other institutional body to which data requests may be sent.

**Response:** We appreciate your concern about the ethical concerns of this work. All ethical considerations have been included in the "Ethical consideration and consent to participate" section of the "Revised manuscript with track changes," Line 234-237, Page 9. In addition, we have submitted the ethics approval that we received from the DHS office as supporting Information **(S1File).**

**3.2.** If there are no restrictions, please upload the minimal anonymized data set necessary to replicate your study findings as either Supporting Information files or to a stable, public repository and provide us with the relevant URLs, DOIs, or accession numbers.

**Response:** Thank you for your inquiry, but sharing the data is not advised. This was explicitly stated in DHS's ethics approval: "Be aware that re-distribution of any DHS micro-level data, either directly or through any tool/dashboard, is not permitted."

**Requirement 4.** Please review your reference list to ensure that it is complete and correct. If you have cited papers that have been retracted, please include the rationale for doing so in the manuscript text, or remove these references and replace them with relevant current references. Any changes to the reference list should be mentioned in the rebuttal letter that accompanies your revised manuscript. If you need to cite a retracted article, indicate the article’s retracted status in the References list and also include a citation and full reference for the retraction notice.

**Response:** Thank you for providing us with the opportunity to revise our work by incorporating all of the valuable comments and suggestions made by the reviewers. We have checked all the references and we assured you that all of them were well-updated and non of them were retracted.

**Additional Editor Comments**

**Comment 1:** The conclusion drawn seems shallow.Better to revise the conclusion. Basically, the recommendations and conclusion have to be drawn from the main finding of the study and it has to be specific enough

**Response:** thank you for your meticulous review. We have re-checked the Conclusion and Recommendation sections and we have corrected the statement by focusing on the findings from the analyses. The corrected version is highlighted in the “Abstract” section of the “Revised manuscript with track changes”, Line 56-61, Page 2.

_____________________________END________________________________________

THANK YOU!!!

**AUTHORS’ RESPONSE TO REVIEWERS**

**Dear: Reviewers**

We thank you for a thorough reading and constructive comments and suggestions on our manuscript and for the opportunity to revise and resubmit. We are pleased to submit the improved research article **“Compliance towards WHO recommendations on antenatal care for a positive pregnancy experience: Timeliness and adequacy of antenatal care visit in Sub-Saharan African countries: Evidence from the most recent standardized Demographic Health Survey data”** for your consideration in the special collection of Plos One. On the following pages, you will find our response to the reviewers’ comments. On behalf of my co-authors, I thank you for your consideration of this resubmission. We appreciate your time and look forward to our responses to the comments and suggestions raised from your side independently as “Authors’ response to Reviewer#1”.

Sincerely,

Aklilu H. (MPH)(corresponding author)

[**aklilihabte57@gmail.com**](mailto:aklilihabte57@gmail.com)

**Authors’ Response to Reviewer**#**1**

**General comments**

**Comment 1:** Dear Editorial Team and Authors: You asked me to review this study titled "Compliance towards WHO recommendations on antenatal care for a positive pregnancy experience: timeliness and adequacy of antenatal care visits in Sub-Saharan African countries: Evidence from the most recent standardized Demographic Health Survey data," and I am so grateful for the opportunity. The issue is intriguing. However, it comes with a lot of limitations. I will be glad to suggest the paper's publication if the author attempts to address the following topics. I am so very excited during reviewing this manuscript. Despite that I have attached some of the comments that should be addressed below. You can download it and try to correct it carefully.

**Response:** Thank you very much for taking the time to review our work and for your positive feedback. We received your thoughtful, and generous review, along with helpful feedback and suggestions, as a valuable contribution to our ongoing work. We have tried to address all the possible comments and suggestions raised by you in the following session.

**Comment 1:** There are many grammatical errors in the manuscript, which is recommended to edit for the English language with regard to grammar, punctuation, spelling, line spacing, and clarity and needs correction

**Response**: We appreciate your thorough review and the opportunity to correct some grammatical errors throughout the manuscript. As per your suggestion, we communicated with some of the language experts in the School of Language and Literature and attempted to address all of your concerns.

**Comment 2:** Try to avoid abbreviations/acronyms from the abstract section. Before writing the full word, don’t use them also. E.g. DHS

**Response:** We have corrected all the inconsistencies and highlighted them in the “Abstract” section of the “Revised manuscript with track changes”.

**Comment 3:** What is the importance of dealing adequate and timely initiation of ANC rather than studying inadequate and delayed/late initiation of ANC? What it implies at the end? **Response:** Thank you very much for your insightful and rational inquiry. Of course, focusing on the inadequate is important, but by studying the level of adequate and timely ANC, we can make recommendations based on the identified determinants that hinder or enhance service uptake. That is why all of our recommendations were focused on the efforts to enhance the uptake of the services.

**Comment 4:** The title should Contain as few words as much possible. But your title is broad and not easy to understand, so make it prices, short and clear.

**Response:** Thank you for your feedback; we have made the necessary changes and attempted to shorten the title and we highlighted in the "Title page" of the "Revised manuscript with track changes", Line 1-3, Page 1

**Comment 5:** There is an inconsistency of included data between title and method sessions; from 2016 to 2021 and from 2016 to 2020

**Response:** Thank you for your thorough review. The correct one is from "2016 to 2021," and it has been corrected throughout the manuscript.

**Comment 6:** The study was conducted in sub-Saharan African countries and there are more than 46 countries here, so why you were including only 18 SSA countries

**Response:** Thank you for inquiring. Most previous studies were conducted prior to 2016, and we were eager to yield a figure that reflected the most recent status of service uptake. Aside from that, the "WHO Recommendations for a positive pregnancy experience" were launched in 2016, and knowing the statistics on the ground may shed light on how to strengthen efforts to implement the service. Only eighteen of the 46 SSA countries had the most recent reports at the time of our request to the DHS office.

**Comment 7:** Please don’t start a word with a capital letter in the middle of a sentence. E.g. Community-level poverty, (in paragraph 3 line 3 of the discussion section).

**Response:** thank you for your in-depth review. We have corrected and highlighted it accordingly in the "Abstract" section of the "Revised manuscript with track changes", Line 48, Page 2

**Comment 8:** In fact, my big concern is what is worth this study more than other similar studies like a study conducted in Ethiopia and other SSA countries with the same issue. in addition, there is no implementation of the new WHO ANC recommendation launched in 2016 in our setup. So what is the relevance of this study?

**Response:** Thank you for inquiring. Although the "WHO Recommendations for a positive pregnancy experience" were launched in 2016, knowing the statistics on the ground may shed light on how to strengthen efforts to implement the service. Working on the study's findings would be an input for further service improvement. Aside from that, almost all countries are currently on their way to implementing this new policy.

**Comment 8:** You were selecting the final model based on AIC, which was the fourth one with the lowest AIC. Why you didn’t consider Deviance for selecting best model fit.

**Response:** Thank you for your inquiry. Of course, the best model can be chosen in any of the above alternatives. The final (Full) model, in this study, was with lowest AIC, BIC, and also deviance (226, 026.4). So, this doesn’t make any change in the selection of the desired model for the study. We have highlighted all the Random Effect and model fitness statistics in Tables 6 and 7 of the "Revised manuscript with track changes", Pages 17&20

**Comment 9:** In the result part in Determinants of adequate ANC visits among women “the likelihood of receiving adequate ANC visits was 3,63 times higher” what does it mean? try to correct it.

**Response:** It was to mean 3.63 and we have corrected and highlighted it in the result section of the "Revised manuscript with track changes", Page 18, Line 368

**Comment 10:** What is the standard or bases for classifying ANC to adequate and in adequate especially in LMIC including Ethiopia the new ANC recommendation was not applied till now?

**Response:** In previous years, the adequacy of ANC was measured in terms of visit frequency. Prior to the newly recommended ANC visit, an adequate visit was assured when a woman received four or more visits all through Focused antenatal care (FANC). However, in the context of this study, adequacy is operationalized by women who received at least eight visits. In terms of implementation, almost all countries, including Ethiopia, have begun to implement the new recommendation.

Comment 12: In your conclusion part Why you say low coverage of the two outcome of interest? What is the base for saying that?

**Response:** In order to meet the SDG recommendation of ending preventable maternal mortality (EPMM), the majority of women must be enrolled in the continuum of maternal health care, and those figures were extremely low when compared to some primary studies and those in developed countries. From the point of our general knowledge, the majority of maternal and child health services in the SSA region were underutilized and this study also confirmed that.

Thank you for your constructive feedback and suggestions, which are valuable in the improvement of our manuscript!!!

**__________________________________________________________________________________________________________END_______________________________________**

**THANK YOU!!!**
